# Substitute for Calomel

**Published:** 1878-08

**Authors:** 


A Substitute for Calomel.—Sulphate of manganese, according to Dr. Goolden, in the London Lancet of June 15th, 1878, is a most excellent substitute for mercury in the various bilious troubles. In jaundice, hepatic dropsy, and hypochondriasis it has produced most remarkable results, and in hemorrhoids and in congestion of the fauces and bronchia it has proved no less efficacious. Anæmic patients who cannot take any of the preparations of iron are enabled to take iron with benefit if combined with two to five grains of sulphate of manganese. Its taste is not unlike that of epsom salts, but it is less bitter. Dr. Goolden prefers to administer the manganese in ten grains to a scruple dose, in a glass of water, adding a little citrate of magnesia to cause effervescence. By these doses large bilious dejections are produced. Half a drachm is the utmost dose ever necessary, and ten grains is usually quite sufficient. The larger doses sometimes produce decided, though temporary nausea, and this may be avoided by adding a small quantity of epsom salt. Its action is attended by neither griping nor depression; neither the heart's action nor the pulse is altered.

Dr. Goolden has employed this medicine freely in private and hospital practice for more than thirty-five years. *Medical Brief.*

